# The *Chromobacterium violaceum* ArsR Arsenite Repressor Exerts Tighter Control on Its Cognate Promoter Than the *Escherichia coli* System

**DOI:** 10.3389/fmicb.2016.01851

**Published:** 2016-11-21

**Authors:** Letícia M. Arruda, Lummy M. O. Monteiro, Rafael Silva-Rocha

**Affiliations:** Systems and Synthetic Biology Lab, Department of Cell and Molecular Biology, Ribeirão Preto Medical School, University of São PauloRibeirão Preto, Brazil

**Keywords:** regulatory network, arsenic response system, *cis*-regulatory elements, *ars* operon, ArsR/SmtB family

## Abstract

Environmental bacteria are endowed with several regulatory systems that have potential applications in biotechnology. In this report, we characterize the arsenic biosensing features of the *ars* response system from *Chromobacterium violaceum* in the heterologous host *Escherichia coli*. We show that the native *Pars/arsR* system of *C. violaceum* outperforms the chromosomal *ars* copy of *E. coli* when exposed to micromolar concentrations of arsenite. To understand the molecular basis of this phenomenon, we analyzed the interaction between ArsR regulators and their promoter target sites as well as induction of the system at saturating concentrations of the regulators. *In vivo* titration experiments indicate that ArsR from *C. violaceum* has stronger binding affinity for its target promoter than the regulator from *E. coli* does. Additionally, arsenite induction experiments at saturating regulator concentration demonstrates that although the *Pars/arsR* system from *E. coli* displays a gradual response to increasing concentration of the inducer, the system from *C. violaceum* has a steeper response with a stronger promoter induction after a given arsenite threshold. Taken together, these data demonstrate the characterization of a novel arsenic response element from an environmental bacterium with potentially enhanced performance that could be further explored for the construction of an arsenic biosensor.

## Introduction

Bacteria that thrive in environments contaminated with toxic compounds are usually endowed with diverse molecular mechanisms related to tolerance to these chemicals (Top and Springael, 2003; Permina et al., 2006). From a physiological point of view, mechanisms for resistance to stressors are usually energy dependent and should be tightly regulated in order to avoid wasting valuable resources when stressors are absent (Nojiri et al., 2004; Diaz et al., 2013). Many bacteria have evolved molecular mechanisms to handle exposure to toxic metals and metalloids. The genes encoding such systems are usually transcriptionally controlled by a plethora of regulatory systems that coordinate gene expression in response to the presence of the cognate chemical.

Several regulatory systems dedicated to the sensing of toxic metals and metalloids have been characterized in bacteria (Busenlehner et al., 2003; Kaur et al., 2006; Permina et al., 2006; Reyes-Caballero et al., 2011). Genomic studies have demonstrated that most of these systems are broadly distributed among different bacterial phyla (Paez-Espino et al., 2009). For instance, transcription factors belonging to the SmtB/ArsR family have been expensively characterized for their role in sensing and controlling gene expression in response to divalent metals (e.g., zinc, nickel, and cadmium) or toxic metalloids (e.g., arsenic and antimonite) (Busenlehner et al., 2003; Eicken et al., 2003; Qin et al., 2007). Members of this family are usually small (~100 aa) regulatory proteins that repress gene expression by blocking access of RNA polymerase to target promoters in the absence of the cognate inducer (VanZile et al., 2000; Kar et al., 2001; Morita et al., 2001; Cavet et al., 2002). These proteins usually act as dimers that bind to target DNA sequences in the apo form. Once in complex with their specific target metal/metalloid, the regulators strongly decrease their affinity for DNA allowing dissociation from the promoter and subsequently, gene expression activation (Kar et al., 1997; Morita et al., 2002, 2003; Eicken et al., 2003; Chauhan et al., 2009).

Understanding the molecular mechanisms behind the transcriptional response to toxic metals and metalloids in bacteria has led to a growing interest in repurposing these systems to construct biosensors for detection of these chemicals in the environment (Stocker et al., 2003; Siddiki et al., 2011; Cortés-Salazar et al., 2013; Chen et al., 2014). Such efforts have been made for the detection of arsenic, a highly abundant and extremely toxic metalloid released to the environment as a result of anthropogenic activity (Matschullat, 2000; Mandal and Suzuki, 2002). In comparison to analytical chemistry methods, biosensors (which encompass a biological sensing component and easily detectable output) would provide reliable, specific, and an inexpensive means for the *in situ* detection of target compounds in environmental samples (Baeumner, 2003).

Constructed biosensors for environmental purposes have generally coupled well-characterized components (usually from bacteria) that are responsive to the target compound with a reporter gene that gives rise to a colorimetric, luminescent, or fluorescent output. In this sense, developed biosensors proved to be useful tools for arsenite and arsenate detection in groundwater (Siegfried et al., 2012) and river water (Siegfried et al., 2015) as a low cost, suitable and transportable alternative to detect the metalloid to prevent or diminish arsenic exposure. Furthermore, the advent of biological circuit design approaches in the field of synthetic biology has allowed the re-wiring of basic molecular components (regulators, DNA binding sites, and operators), which can be reinserted into the host cell to give rise to sensors with enhanced performance (Stocker et al., 2003; Trang et al., 2005; Fernandez et al., 2016; Merulla and van der Meer, 2016). In fact, the addition of ArsR operator downstream of its target promoter generates reduction in the background noise, which reduces the detection limits to as lower as one microgram per liter (Merulla and van der Meer, 2016).

Although changing the circuit design can improve the efficiency of the biosensor, the utilization of molecular components with intrinsically enhanced transcriptional performance when induced with the target compound could lead to a system with superior behavior. With this reasoning in mind, we aimed to identify a natural system that displayed enhanced transcriptional response to arsenite (As^III^). We focused on the *ars* system from *Chromobacterium violaceum*, a gram-negative bacterium with a low arsenic tolerance level but endowed with a fully functional *Pars* and ArsR regulatory system (Azevedo et al., 2008; Silva-Rocha et al., 2013a). We demonstrate that the *ars* system from *C. violaceum* has superior arsenic induction performance when compared to the chromosomal prototype system in *Escherichia coli* and that these differences can be traced to the binding affinity of ArsR regulators to their DNA targets and to the occurrence of a stronger transcriptional response under inducing conditions in the *C. violaceum* system. Taken together, the results shown here demonstrate the potential of environmental bacteria as a reservoir of molecular components with enhanced performance for biosensor design, as well as a characterization of a novel arsenic ArsR transcription factor in bacteria.

## Materials and Methods

### Bacterial Strains, Plasmids, and Growth Conditions

Bacterial strains, plasmids, and primers used in this study are listed in **Table 1**. *E. coli* DH5α cells were used for cloning procedures. *E. coli* W3110 was used as the wild type strain, whereas *E. coli* AW3110 (Δ*ars* operon) was used as the mutant host for testing the circuits. *E. coli* strains were grown at 37°C in LB media (Sambrook et al., 1989) or M9 minimal media (6.4 g/L Na2HPO4⋅7H2O, 1.5 g/L KH2PO4, 0.25 g/L NaCl, and 0.5 g/L NH4Cl) supplemented with 2 mM MgSO_4_, 0.1 mM casamino acid, and 1% glycerol as the sole carbon source. When required, kanamycin (Km, 50 μg/mL) or chloramphenicol (34 μg/mL) was added to the media to ensure plasmid retention. When cells were grown in minimal media, antibiotics were used at half concentrations. For induction experiments, benzoic acid (Sigma–Aldrich, St. Louis, MO, USA) and sodium arsenite (Sigma–Aldrich) were used at different concentrations.

**Table 1:** Strains, plasmids, and primers used in this study.

Strains, plasmids, and primers	Description	Reference
**Strains**		
*Chromobacterium violaceum ATCC12472*	Wild type strain of *C. violaceum*	ATCC collection
*Escherichia coli* DH5α	*F^–^ φ80*Δ*lacZ*Δ*M15*Δ*(lacZYAA-argF) U169 recA1 endA1 hsdR17 R^–^M^+^ supE4 thi gyrA relA*	Sambrook et al., 1989
*E. coli* W3110	*K12 F^–^ IN (rrnD-rrnE)*	Bachmann, 1972
*E. coli* AW3110	*K12 F^–^*Δ*ars::cam IN (rrnD-rrnE)*, *ars* mutant strain	Carlin et al., 1995
**Plasmids**		
pMR1	Cm^R^; orip15a; promoter probe variant of pRV2	Guazzaroni and Silva-Rocha, 2014
pMR1-*Pars_cvi_*::*arsR_cvi_*	Cm^R^; pMR1 inserted with the operator, promoter and regulator region as native arrangement from *C. violaceum*	This study
pMR1-*Pars_eco_*::*arsR_eco_*	Cm^R^; pMR1 inserted with the operator, promoter and regulator region as native arrangement from *E. coli*	This study
pRV2	Km^R^, orip15a; dual promoter probe vector with GFPlva and mCherry reporters	Silva-Rocha and de Lorenzo, 2011
pRV2-*Pars_cvi_*	Km^R^, *Pars_cvi_* –GFPlva transcriptional fusion	This study
pRV2-*Pars_eco_*	Km^R^, *Pars_eco_* -GFPlva transcriptional fusion	This study
pSEVA438	Sm^R^, ori pBBR1; expression vector based on the benzoate inducible *xylS-Pm* system	Silva-Rocha et al., 2013b
pSEVA438-*arsR_cvi_*	Sm^R^, pSEVA438 inserted with *arsR_cvi_* gene	This study
pSEVA438-*arsR_eco_*	Sm^R^, pSEVA438 inserted with *arsR_eco_* gene	This study
**Primers^∗^**		
5arsecEco	GCGCGAATTCCCGCCAGCTGAAGAAATCG	Thisstudy
3arsecBam	GCGCGGATCCCCAGTAACATAATGCCTCCC	This study
5arscvEco	GCGTGAATTCGTAGTTCGGC	This study
3arscvBam	GCGCGGATCCCTGCTTCAGCCAGGATGG	This study
5arsRcvEco	CGCGGAATTCAGGAGGAAAAACATATGGAAATGAAAAGTGCTGT	This study
3ParscvBam	CGCGGATCCGACTTAACGAATGTGTAAGTGC	This study
5arsRecEco	CGCGGAATTCAGGAGGAAAAACATATGTTTTTGACTTATCCGCTTCG	This study
3ParsecBam	GCGCGGATCCCAAGAGCGTGACAGCAC	This study

*^∗^Restriction sites are underlined in the primer sequence.*

### Plasmid and Strain Construction

For analysis of the *ars* system under negative feedback, DNA fragments containing the *Pars/arsR* elements from *C. violaceum* and *E. coli* were PCR amplified from genomic DNA using primers 5arscvEco/3arscvBam and 5arsecEco/3arsecBam, respectively (**Table 1**). DNA fragments were amplified using Phusion High-Fidelity DNA polymerase (New England Biolabs, Ipswich, MA, USA) according to the manufacturer’s protocol. The resulting DNA fragments were cloned into the pMR1 reporter vector (Guazzaroni and Silva-Rocha, 2014), which carries the *gfplva*, a short lived variant of GFP. The resulting recombinant plasmids were named pMR1-*Pars_cvi_*::ArsR*_cvi_* and pMR1-*Pars_eco_*::ArsR*_eco_* (**Table 1**; Supplementary Material). The correct DNA sequence was verified by sequencing using the dideoxy terminal method. Next, the recombinant plasmids were introduced into *E. coli* W3110 using chemically competent cells (Sambrook et al., 1989). The resulting reporter strains were used for induction experiments. For cloning and analysis of individual promoters, *Pars_cvi_* and *Pars_eco_* were PCR amplified using primers 5arscvEco/3ParscvBam and 5arsecEco/3ParsecBam, gel purified, and cloned into pRV2 using E*co*RI/*Bam*HI restriction sites. The resulting plasmids, pRV2-*Pars_cvi_* and pRV2-*Pars_eco_*, were transformed into *E. coli* W3110 (wild type) and *E. coli* AW3110 (mutant) strains. To construct the uncoupled circuit, the *arsR* genes from each organism were cloned under the control of the XylS/*Pm* system, which is inducible by benzoate (Blatny et al., 1997). Each regulator was amplified using primers 5arsRcvEco/3arscvBam and 5arsRecEco/3arsecBam (forward primers introduced a strong RBS sequence at the 5′ region of the gene) and resulting fragments were cloned into pSEVA438 (Silva-Rocha et al., 2013b) using *Eco*RI/*Bam*HI enzymes. The resulting plasmids were named pSEVA438-*arsR_cvi_* and pSEVA438-*arsR_eco_* and co-transformed along with the cognate GFP reporter plasmid (pRV2-*Pars_cvi_* or pRV2-*Pars_eco_*) into *E. coli* AW3110 to generate the reporter strains.

### GFP Fluorescence Assay and Data Processing

To measure promoter activity, freshly plated single colonies were grown overnight in LB media, washed, and resuspended in fresh M9 media. Ten microliters of culture was assayed in 96-well microplates in biological triplicate with 170 μL of M9 media supplemented with required antibiotics and different As^III^ or benzoate concentrations. Cell growth and GFP fluorescence was quantified using a Victor X3 plate reader (PerkinElmer, Waltham, MA, USA) and calculated as arbitrary units by dividing the fluorescence level by the optical density at 600 nm (reported as GFP/OD_600_) after background correction. Background signal was evaluated with the same strain harboring the pMR1 (Guazzaroni and Silva-Rocha, 2014) or pRV2 (Silva-Rocha and de Lorenzo, 2011) empty plasmid. Unless otherwise indicated, measurements were taken at 30 min intervals over 8 h. All experiments were performed at least three times. Raw data were processed using *ad hoc* R script^1^) and plots were constructed using Microsoft Excel, R, or MeV^2^.

## Results

### The Natural *ars* System from *C. violaceum* Displays Enhanced Induction by As^III^

**FIGURE 1:**
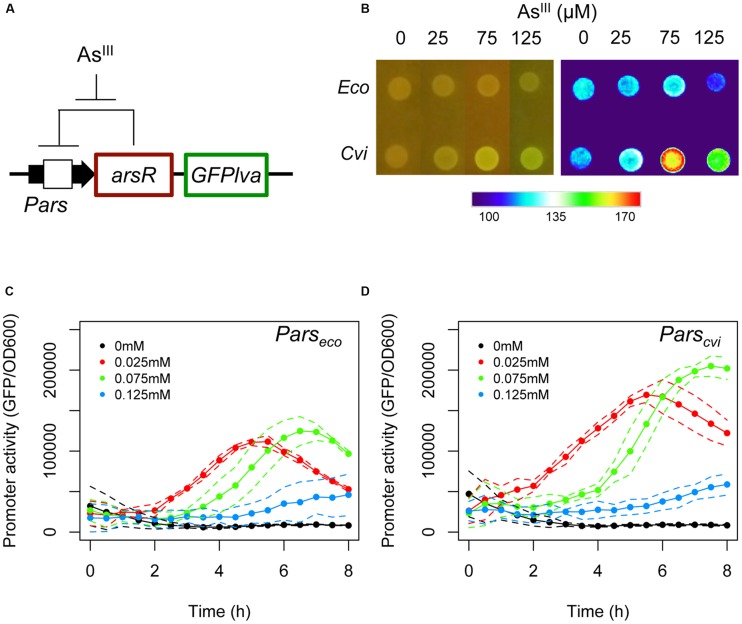
**Analysis of the natural *ars* circuits from *Escherichia coli* and *Chromobacterium violaceum*. (A)** Schematic representation of the feedback circuit controlling GFPlva expression. **(B)** Induction of the *ars* system from *E. coli* and *C. violaceum* on agar plates using increasing concentrations of As^III^ for 6 h. The left panel represents bacterial colonies grown in agar plates and at the right panel the GFP intensities were converted to a false color scale to facilitate the visualization of the differences. **(C,D)** Induction kinetics of the *ars* system from *E. coli* **(C)** and *C. violaceum* **(D**) in wild type (W3110) *E. coli* in M9 liquid media exposed to increasing concentrations of As^III^. Solid lines represent the average of three independent experiments, whereas dashed lines represents the upper and lower limits of standard deviations.

In order to characterize the arsenic response system, we initially focused on the response system from *C. violaceum*, an environmental bacterium with low tolerance to arsenic (Azevedo et al., 2008; Silva-Rocha et al., 2013a). Because this bacterium is sensitive to micromolar doses of As^III^, we reasoned that in the natural environment, this organism would have to trigger a strong transcriptional response to very low concentrations of this metalloid and thus, would be endowed with an intrinsically sensitive *ars* response system. In order to compare the performance of the *ars* system from *C. violaceum* with that of *E. coli* (the prototype system used for arsenic biosensor construction), we cloned the *Pars/arsR* elements from both organisms into a GFP reporter vector and introduced the resulting construct into *E. coli*. This allowed us to faithfully assess the response of the native systems (i.e., retaining the negative feedback loop, **Figure 1A**) in the same bacterial host.

As seen in **Figure 1B**, the *Pars/arsR* system from *C. violaceum* had a higher GFP output upon exposure to 75 μM of As^III^ when assayed on agar plates compared to the *E. coli* system. In order to analyze the two systems quantitatively, we performed induction experiments in liquid minimal media and quantified GFP production at fixed time intervals. When we compared the performance of the two systems, we observed that the *Pars/arsR* genes from *C. violaceum* displayed GFP induction dynamics significantly higher then that from *E. coli* (**Figures 1C,D**). It is worth mentioning that the concentration of 125 μM produces a reduced output due to the strong toxicity of arsenite to the strain. This result confirms our hypothesis that the *Pars/arsR* system from *C. violaceum* has a more efficient transcriptional response to As^III^. Taking into account the architecture of the *ars* regulatory elements, the results observed in **Figure 1** could be due to differences in three parameters of the two systems. First, the *Pars_cvi_* promoter could have a stronger intrinsic activity than *Pars_eco_*. In this scenario, releasing ArsR repression would allow increased promoter activity at *Pars_cvi_*. Second, ArsR_cvi_ could have stronger binding affinity by its target DNA sequence than ArsR_eco_. If that were the case, the feedback loop would stabilize in lower amounts of ArsR in *C. violaceum*, which could be easily inactivated by changes in As^III^ concentrations, leading to higher promoter output. Finally, ArsR_cvi_ could have higher affinity for As^III^ and thus, small changes in concentration of the inducer would lead to increased inactivation of the repressor, resulting in higher promoter activity. In order to investigate these possibilities, we conducted a number of *in vivo* experiments to quantify the relative promoter strengths, the apparent repressor-promoter binding affinities, and the repressor-effector interactions as described in next sections.

### *Pars_cvi_* and *Pars_eco_* Have Similar Strengths with Different Kinetics in *E. coli*

**FIGURE 2:**
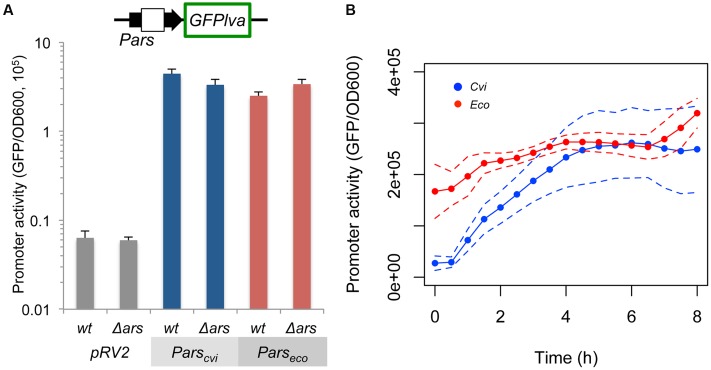
**Quantification of the intrinsic *Pars_cvi_* and *Pars_eco_* activities.** Wild type (wt) *E. coli* or mutant (Δ*ars*) strains harboring the reporter plasmids containing the GFPlva gene under the control of the specific promoters were assayed in M9 media in the absence of As^III^. Empty pRV2 plasmid was used as control. **(A)** Maximal activity of the specific promoter after 6 h of growth. **(B)** Promoter dynamics during growth of *E. coli* AW3110 (Δ*ars*) harboring pRV2-*Pars_cvi_* (Cvi, blue) or pRV2-*Pars_eco_* (Eco, red). Solid lines represent the average of three independent experiments, whereas dashed lines represents the upper and lower limits of standard deviations.

In order to understand the molecular differences leading to the observed behavior of the *ars* systems from *C. violaceum* and *E. coli*, we first compared the relative promoter strengths in the absence of repression. For this experiment, *Pars_cvi_* and *Pars_eco_* were cloned upstream of a GFP*lva* reporter gene and introduced into wild type and *ars* mutant strains of *E. coli*. As shown in **Figure 2A**, both promoters displayed similar maximal activities in wild type and mutant *E. coli*, indicating that promoter activity alone could not explain the differences observed in **Figure 1**. When we compared promoter dynamics alongside the growth curve, we observed that *Pars_eco_* displays high initial activity that tends to stabilize after 2 h of growth, whereas *Pars_cvi_* activity increases dramatically in the first 4 h and then reaches a similar steady-state level (**Figure 2B**).

### The ArsR_cvi_ Regulator Has a Higher Apparent Affinity for Its Target Promoter

**FIGURE 3:**
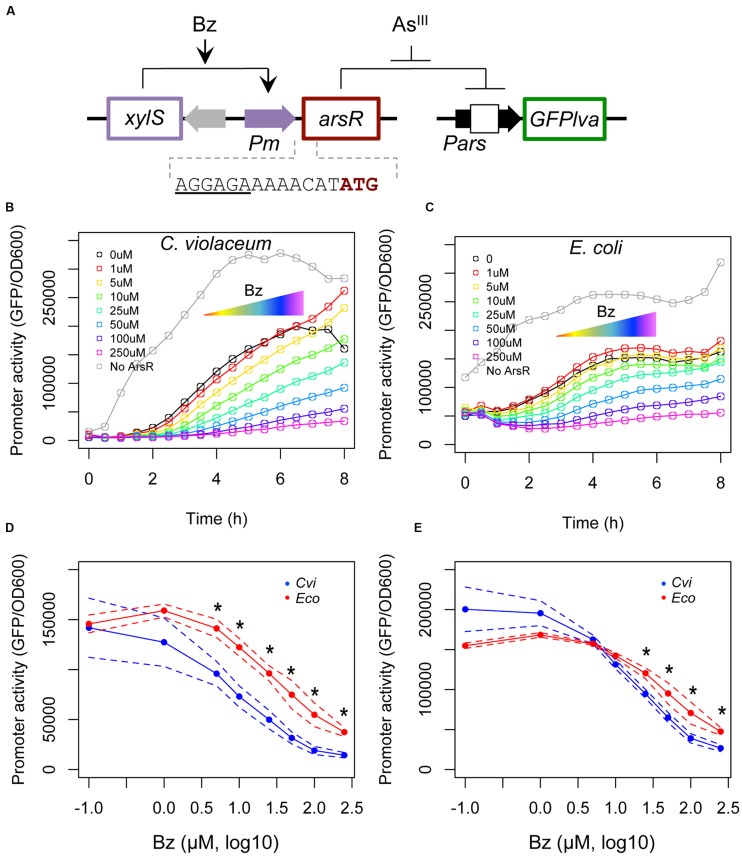
***In vivo* titration experiments using a benzoate inducible system. (A)** Schematic representation of the circuit used. In this system, the target regulator is placed under the control of the XylS/*Pm* expression cassette from pSEVA438 (Silva-Rocha et al., 2013b). To ensure similar levels of ArsR regulators were produced, a strong RBS element was introduced between the *Pm* promoter and the start codon of the gene. A second plasmid based on pRV2 was used where expression of a GFPlva reporter gene is placed under the control of the target promoter. Promoter activities were assayed during the growth curve analysis using increasing concentrations of benzoate (Bz, from 0 to 250 μM). **(B)** *In vivo* titration of ArsR/*Pars* from *C. violaceum*. **(C)** *In vivo* titration of ArsR/*Pars* from *E. coli*. Gray lines represent control strains harboring the empty pSEVA438 vector (i.e., no ArsR production). **(D,E)** Repression kinetics of *Pars_cvi_* (blue) and *Pars_eco_* (red) upon exposure to increasing benzoate concentrations after four **(D)** or six **(E)** hours of induction. Solid lines represent the average of three independent experiments, whereas dashed lines represents the upper and lower limits of standard deviations. Statistics differences are highlighted by (^∗^) as analyzed using Student’s *t*-test with *p*-value *p* < 0.05.

Once we determined the maximal transcriptional activities of both systems, we investigated the effect of increasing concentrations of ArsR on the activity of *Pars_cvi_* and *Pars_eco_* promoters. We constructed an uncoupled inducible system (**Figure 3A**) where ArsR production is under the control of a benzoate inducible system based on the XylS/*Pm* element (Blatny et al., 1997). The specific *Pars* promoter is fused to a GFP reporter gene in order to provide a fluorescence output. In this system, the RBS sequences for both regulators are changed to the *tir* element, thus ensuring similar translation rates for both proteins. Using this setup, we induced the cells with increasing concentrations of benzoate to generate a stepwise decrease in promoter outputs (**Figures 3B,C**) (Brewster et al., 2014). Assuming that both ArsR_eco_ and ArsR_cvi_ are produced from the same transcription and translation signals, the protein level produced should be equivalent and thus, should allow us to indirectly infer the binding affinity of each repressor for its target DNA sequence, similarly to the approach used by Wang and coworkers (Wang et al., 2015). The results are shown in **Figures 3D,E**, which represents the effect of increasing benzoate concentrations on target promoter activity at 4 and 6 h, respectively. These *in vivo* titration experiments indicate that *Pars_cvi_* has a higher decay in activity with increasing benzoate concentration. These results show that ArsR_cvi_ has a higher *in vivo* affinity for its target DNA. By comparing the benzoate concentration required to reduce promoter activity in each system, we find that approximately five times more inducer is required to reduce *Pars_eco_* activity than *Pars_cvi_* activity (62 μM vs. 12 μM).

### The *ars* System from *C. violaceum* Shows Stronger Transcriptional Response to Arsenic

**FIGURE 4:**
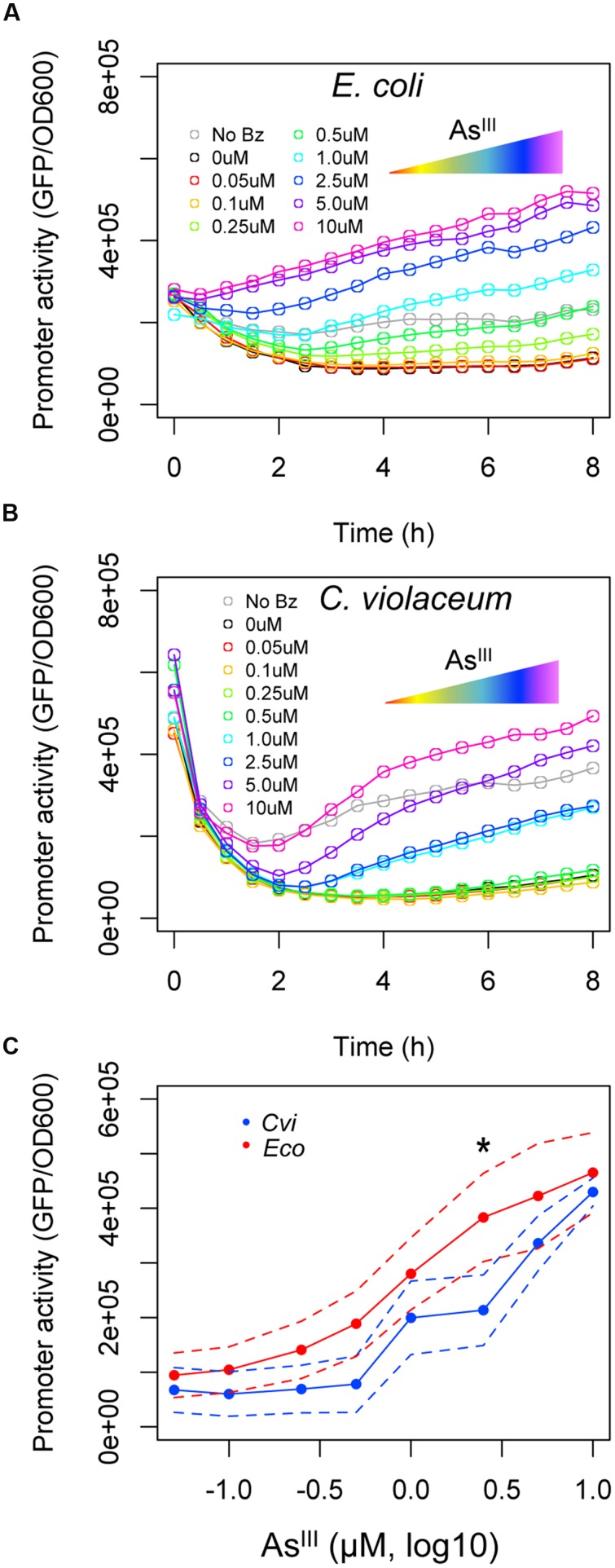
**Induction of *ars* systems with increasing concentrations of As^III^.** *E. coli* AW3110 (Δ*ars*) harboring the circuits represented in **Figure 3A** were exposed to a saturating concentration of benzoate (250 μM) and varying concentrations of As^III^ (from 0 to 10 μM). **(A)** The expression profile of the *ars* system from *E. coli* induced with increasing concentrations of As^III^. **(B)** The expression profile of the *ars* system from *C. violaceum* induced with increasing concentrations of As^III^. **(C)** Induction kinetics of *Pars_cvi_* (blue) and *Pars_eco_* (red) upon exposure to increasing As^III^ concentrations after 6 h of induction. Solid lines represent the average of three independent experiments, whereas dashed lines represents the upper and lower limits of standard deviations. Statistics differences are highlighted by (^∗^) as analyzed using Student’s *t*-test with *p*-value *p* < 0.05.

**FIGURE 5:**
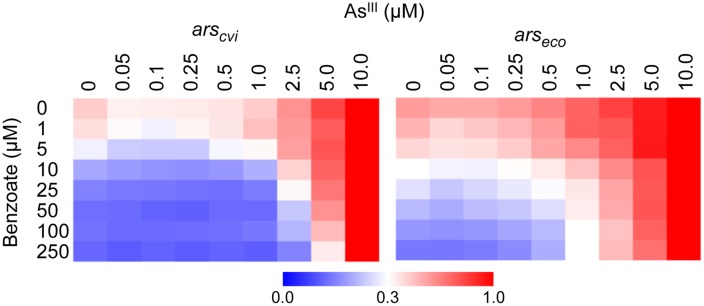
**Expression dynamics of *ars* systems from *E. coli* and *C. violaceum* under varying concentrations of benzoate and As^III^.** *E. coli* AW3110 (Δ*ars*) harboring the circuits represented in **Figure 3A** were exposed to varying concentrations of benzoate and As^III^ and incubated for 6 h to allow induction. Promoter activities (calculated as GFP/OD_600_) were normalized to the maximal level obtained with 10 μM of As^III^. Data are representative of three independent experiments.

Once we observed that the ArsR repressor from *C. violaceum* has a higher apparent affinity for its target than the ArsR repressor from *E. coli*, we analyzed the effect of arsenic binding during allosteric derepression of the system. We used the maximal benzoate concentration (250 μM) to ensure the ArsR concentration was high enough to fully repress *Pars_cvi_* and *Pars_eco_*. Using this setup, we exposed the reporter strain harboring the circuits from **Figure 3A** to increasing concentrations of As^III^. As shown in **Figure 4A**, under saturating regulator concentrations, the *ars* system from *E. coli* is gradually induced with As^III^ concentrations above 0.25 μM. However, under similar conditions, the *ars* system from *C. violaceum* is insensitive to As^III^ concentrations below 1.0 μM (**Figure 4B**). This differential induction behavior could be observed in the dose-response curve for both systems at 6 h post-induction (**Figure 4C**) where the *ars* system from *C. violaceum* is insensitive to As^III^ concentrations below 1.0 μM and displays pronounced induction above this threshold. This remarkable difference between the responses of the systems indicates that the *ars* from *C. violaceum* displays steeper induction kinetics, where the system is completely OFF at low concentrations of inducer but displays strong induction upon reaching a certain threshold. In order to test whether this behavior was the result of the high level of ArsR_cvi_ produced (due to the elevated benzoate concentration used), we performed induction experiments in which we varied both benzoate and As^III^ concentrations. As shown in **Figure 5**, the *ars* system from *E. coli* displayed a gradual induction by As^III^ under varying benzoate concentrations, whereas the induction of the *C. violaceum* system presented steeper slope even at reduced benzoate concentrations (and thus low ArsR_cvi_ levels). Taken together, these results indicate that the differences in the induction profiles of both systems are the result of intrinsic differences in the way the ArsR regulators interact with As^III^.

## Discussion

Bacteria able to thrive in contaminated environments are endowed with very efficient detoxification mechanisms controlled at the gene expression level. In this context, different metalloregulatory proteins have evolved to control gene expression in response to different metals and metalloids. *In vivo* functional characterization of the *ars* system from several bacteria has revealed strong variation in the number of resistance genes, operon organization and, final resistance levels (Carlin et al., 1995; Paez-Espino et al., 2009). However, a conserved feature of the *ars* system is that the first gene of the operon encodes the ArsR regulator, which repress its own expression in a feedback loop (Wu and Rosen, 1993; Xu and Rosen, 1997). The ArsR from both *E. coli* and *C. violaceum* are members of the SmtB/ArsR family. This class of transcription factors include small proteins that share a core secondary structure formed by five alpha helixes and two anti-parallel beta strands in the form ααααββα (Cook et al., 1998; Busenlehner et al., 2003). Although DNA binding occurs through the helix-turn-helix domain formed by helix three and four, members of this family have a more degenerate inducer-binding site that could be located in helix three (Type 1 regulators – ArsR_eco_) or in helix five at the dimerization interface of the protein [Type 2 regulators – ArsR_cvi_ (Qin et al., 2007)]. Considering the metal binding site, ArsR proteins have a conserved metalloid-protein interaction interface, in which cysteine residues are required to coordinate the ligand (Busenlehner et al., 2003). ArsR_eco_ has an As^III^ binding site formed by Cys residues located at helix three (Wu and Rosen, 1993), whereas ArsR_cvi_ belongs to the class of regulators where binding is formed by Cys residues at helix five (Qin et al., 2007; Azevedo et al., 2008). These differences between the two classes of regulators also imposes differences on the intramolecular allosteric switch upon inducer binding because type 1 regulators have the ligand-binding site close to the DNA binding domain, whereas signal transmission in type 2 regulators must occur from a distance (Qin et al., 2007).

While differences in the localization of metalloid biding site of the regulatory proteins might account for the different expression behaviors observed, other parameters such as protein-DNA interaction might play an important role in the process. For instance, Li et al. (2015) have used *in vitro* mutagenesis to tune the arsenic response of the *ars* system from the R773 plasmid of *E. coli*. By analyzing the variants with enhanced performance, they found several mutations into the ArsR binding site (ABS) at the DNA. This found is particularly relevant since, while members of the SmtB/ArsR family recognize palindromic DNA sequences, the ABS sequence found in the *ars* promoter of *E. coli* has an imperfect palindromic sequence (Wu and Rosen, 1993). As the *ars* systems from *E. coli* and *C. violaceum* have no conserved ABS sequence (Carepo et al., 2004; Azevedo et al., 2008), this difference on protein-DNA interface may explain the stronger binding affinity of ArsR_cvi_ and the difference in the dynamics of gene expression. Altogether, these structural differences between the two regulators analyzed here could explain the discrepant behavior (i.e., gradient vs. steeper) during expression of the *ars* systems.

While the ArsR from *E. coli* proved to be more sensible to lower arsenic concentrations when assayed in the decoupled system (**Figures 4** and **5**), the *C. violaceum* system displayed a better triggering system at higher concentrations, with a steeper slope promoter induction. Although the particular features of the two classes of regulators have not been systematically investigated, different molecular mechanisms for an allosteric switch induced by As^III^ binding could be the reason for the different induction profiles observed for ArsR regulators of *C. violaceum* and *E. coli*. Uncovering these mechanisms should be a target of future research in order to further understand the evolution of SmtB/ArsR protein family members and for biosensor development.

Microorganisms are a valuable source of molecular components for the construction of biological circuits for biotechnological applications (Cheng and Lu, 2012; Brophy and Voigt, 2014). From a historical perspective, most synthetic circuits constructed in bacteria have been implemented using components from the model organism *E. coli* (Church et al., 2014). This has indeed been the case for biosensors designed to detect arsenic in environmental samples with several designs having been constructed by shuffling the *ars* components of *E. coli* (Siegfried et al., 2012, 2015; Wang et al., 2015; Merulla and van der Meer, 2016). Since the World Health Organization (WHO) recommended acceptable limit for drinking water of 10 μg/L arsenic, any modification of natural arsenic sensing systems for biosensing purposes should aim increasing responsivity of the systems to concentrations close to this limit. The characterization of the *ars* components of an environmentally relevant bacterium, *C. violaceum*, shows that orthologous systems dedicated to the same function (in this case, arsenic detoxification from the cell) could have different dynamic properties. In the case of the *ars* system from *C. violaceum*, increased ArsR binding affinity by the target DNA sequence together with a stronger induction profile makes these components very attractive for novel designs of arsenic biosensing. Additionally, to the best of our knowledge, this is the first report of a naturally occurring arsenic response system in bacteria with such an atypical induction profile, which provides new venues for the investigation of *ars* evolution and for the application of this pathway in biosensor design.

## Author Contributions

RS-R and LA designed the experimental strategy. LA and LM performed the experiments. LA, LM, and RS-R analyzed and interpreted the data. LA and RS-R wrote the manuscript. All authors have read and approved the final version of the manuscript.

## Conflict of Interest Statement

The authors declare that the research was conducted in the absence of any commercial or financial relationships that could be construed as a potential conflict of interest.

## Footnotes

1 https://www.r-project.org/

2 www.tm4.org/mev.html
