# Supplementary material for: The Chromobacterium violaceum ArsR Arsenite Repressor Exerts Tighter Control on Its Cognate Promoter Than the Escherichia coli System
Source: Front Microbiol. 2016 Nov 21;7:1851. doi: 10.3389/fmicb.2016.01851 (PMC5116461; doi:10.3389/fmicb.2016.01851)
Supplement: Supplementary file 1 [file Data_Sheet_1.docx]

Supplementary Material

The *Chromobacterium violaceum* ArsR arsenite repressor exerts tighter control on its cognate promoter than the *Escherichia coli* system

**Letícia Magalhães Arruda, Lummy Maria Oliveira Monteiro, and Rafael Silva-Rocha***

*** Correspondence:** Rafael Silva-Rocha: silvarochar@gmail.com

# Supplementary Data

**Promoters and regulators sequences reported in Table 1.** For all sequences, the primer sequences are highlighted in italic; the restriction sites are underlined; the start and the stop codon are in bold and the RBS_tir_ sequence is in green. The promoter regions are in blue and the open reading frame of ArsR are in red.

*>Pars_cvi_*::*arsR_cvi_*

*GCGTGAATTCGTAGTTCGGC*GGACAGGTCGCCGGCATGGAAAAGCTTTTCGGGGTGAAGACGACTTCGCGCGCGAGCGCGGTCTCCCGAGCCGGTCATTGATACCCTGCGGAGGATCTAGTTCGCACATTTTTCTTGACCGGAGTTTTTATTTCCATAAATATGGAAAT**ATG**GAAATGAAAAGTGCTGTCACGCTCTTGGCCGCCTTGGCGCAGGACACTCGCCTAGCGATTTATCGACTTCTGGTTCAGCAAGGCCCTGAAGGCCTGGCAGTCGGCCAGATCGGAGAACGGCTTGCTGTGGCTAATGCCACGCTGTCCTTTCATTTGAAGGAGCTATCTCATGCCGGGCTGATTTTGTCCCGCCAAGAGGGGCGCTTTATCTACTACTCAGCCAACTACGAGCAGATGAACGCCTTGCTAGGTTTTCTGACCGAAAACTGTTGTCGCGGCGAAACCTGCACGCCAGCAAACAATGTGCCGCCCTGCGATGGCACTTGC**TGA**AAGAGGCGAACTCATCATGAATCAACCTACGGCAAATGTGCTGTTCTTATGCACGGGCAATTCCTGCCGCT*CCATCCTGGCTGAAGCAGGGATCCGCGC*

> *Pars_eco_*::*arsR_eco_*

*GCGCGAATTCCCGCCAGCTGAAGAAATC*GCTAATTCTTGCAATGTTAGCCACTGGCTAATAGTATTGAGCTGTTAGATAAGAACTCTCTCACTCCAGCCAGAGCCACCAACTCAGGGCTGGAAAGTAAAAAACCGACGCAAAGTCGGTTTTTTTACGTCCTGATTCAGACCTCCTTTCAAATGAATAGCCAACTCAAAATTCACACCTATTACCTTCCTCTGCACTTACACATTCGTTAAGTCATAT**ATG**TTTTTGACTTATCCGCTTCGAAGAGAGACACTACCTGCAACAATCAGGAGCGCAATATGTCATTTCTGTTACCCATCCAATTGTTCAAAATTCTTGCTGATGAAACCCGTCTGGGCATCGTTTTACTGCTCAGCGAACTGGGAGAGTTATGCGTCTGCGATCTCTGCACTGCTCTCGACCAGTCGCAGCCCAAGATCTCCCGCCACCTGGCATTGCTGCGTGAAAGCGGGCTATTGCTGGACCGCAAGCAAGGTAAGTGGGTTCATTACCGCTTATCACCGCATATTCCAGCATGGGCGGCGAAAATTATTGATGAGGCCTGGCGATGTGAACAGGAAAAGGTTCAGGCGATTGTCCGCAACCTGGCTCGACAAAACTGTTCCGGGGACAGTAAGAACATTTGCAGTTAAAAATTTAGCTAAACACATA**TGA**ATTTTCAGATGTGTTTTATCC*GGGAGGCATTATGTTACTGGGGATCCGCGC*

> *Pars_cvi_*

*GCGTGAATTCGTAGTTCGGC*GGACAGGTCGCCGGCATGGAAAAGCTTTTCGGGGTGAAGACGACTTCGCGCGCGAGCGCGGTCTCCCGAGCCGGTCATTGATACCCTGCGGAGGATCTAGTTCGCACATTTTTCTTGACCGGAGTTTTTATTTCCATAAATATGGAAAT**ATG**GAAATGAAAA*GTGCTGTCACGCTCTTGGGATCCGCG*

> *Pars_eco_*

*GCGCGAATTCCCGCCAGCTGAAGAAATCG*CTAATTCTTGCAATGTTAGCCACTGGCTAATAGTATTGAGCTGTTAGATAAGAACTCTCTCACTCCAGCCAGAGCCACCAACTCAGGGCTGGAAAGTAAAAAACCGACGCAAAGTCGGTTTTTTTACGTCCTGATTCAGACCTCCTTTCAAATGAATAGCCAACTCAAAATTCACACCTATTACCTTCCTCT*GCACTTACACATTCGTTAAGTCGGATCCCGCG*

> *arsR_cvi_*

*CGCGGAATTCAGGAGGAAAAACAT****ATG****GAAATGAAAAGTGCTGT*CACGCTCTTGGCCGCCTTGGCGCAGGACACTCGCCTAGCGATTTATCGACTTCTGGTTCAGCAAGGCCCTGAAGGCCTGGCAGTCGGCCAGATCGGAGAACGGCTTGCTGTGGCTAATGCCACGCTGTCCTTTCATTTGAAGGAGCTATCTCATGCCGGGCTGATTTTGTCCCGCCAAGAGGGGCGCTTTATCTACTACTCAGCCAACTACGAGCAGATGAACGCCTTGCTAGGTTTTCTGACCGAAAACTGTTGTCGCGGCGAAACCTGCACGCCAGCAAACAATGTGCCGCCCTGCGATGGCACTTGC**TGA**AAGAGGCGAACTCATCATGAATCAACCTACGGCAAATGTGCTGTTCTTATGCACGGGCAATTCCTGCCGCT*CCATCCTGGCTGAAGCAG GGATCCGCGC*

> *arsR_eco_*

*CGCGGAATTCAGGAGGAAAAACAT****ATG****TTTTTGACTTATCCGCTTCG*AAGAGAGACACTACCTGCAACAATCAGGAGCGCAATATGTCATTTCTGTTACCCATCCAATTGTTCAAAATTCTTGCTGATGAAACCCGTCTGGGCATCGTTTTACTGCTCAGCGAACTGGGAGAGTTATGCGTCTGCGATCTCTGCACTGCTCTCGACCAGTCGCAGCCCAAGATCTCCCGCCACCTGGCATTGCTGCGTGAAAGCGGGCTATTGCTGGACCGCAAGCAAGGTAAGTGGGTTCATTACCGCTTATCACCGCATATTCCAGCATGGGCGGCGAAAATTATTGATGAGGCCTGGCGATGTGAACAGGAAAAGGTTCAGGCGATTGTCCGCAACCTGGCTCGACAAAACTGTTCCGGGGACAGTAAGAACATTTGCAGTTAAAAATTTAGCTAAACACATA**TGA**ATTTTCAGATGTGTTTTATCC*GGGAGGCATTATGTTACTGGGGATCCGCGC*

# Supplementary Figures and Tables

## Supplementary Figures

**Supplementary Figure 1.**

**Figure S1.** Reporter plasmid pMR1.
